# A Fast Scoring of Human Primary Respiratory Epithelia Grown at Air–Liquid Interface (ALI) to Assess Epithelial Morphology in Research and Personalized Medicine Settings

**DOI:** 10.3390/jpm14010109

**Published:** 2024-01-18

**Authors:** Christopher T. Lutsch, Longhua Feng, Ana Gómez Hohn, Lennart Brandt, Stephanie Tamm, Sabina Janciauskiene, Frauke Stanke, Danny Jonigk, Anna-Maria Dittrich, Peter Braubach

**Affiliations:** 1 Department of Pediatric Pneumology, Allergology and Neonatology, Hannover Medical School, 30625 Hannover, Germany; 2 Biomedical Research in Endstage and Obstructive Lung Disease Hannover (BREATH), German Center for Lung Research (DZL), 30625 Hannover, Germany; janciauskiene.sabina@mh-hannover.de (S.J.); djonigk@ukaachen.de (D.J.); 3 Institute for Pathology, Hannover Medical School, 30625 Hannover, Germany; 4 Department of Respiratory Medicine, Hannover Medical School, 30625 Hannover, Germany; 5 Institute of Pathology, School of Medicine, University Hospital RWTH Aachen, 52074 Aachen, Germany

**Keywords:** primary respiratory epithelia, air–liquid interface culture, morphology score, goblet cells

## Abstract

Background: In recent years, increasingly complex ALI protocols involving specialized, albeit laboratory-specific media have been established, while at the same time, many studies compile the data of only a few ALI donors in spite of site-, protocol- and donor-specific differentiation. Methods: We describe a simple morphology scoring protocol using histology material derived from epithelia grown on ALI inserts in parallel to other, more complex readouts. Results: Among more than 100 ALI inserts derived from different donors, significant differences in layer score (*p* = 0.001) and goblet cell score (*p* = 0.002) were observed when ALI epithelia derived from explanted lung material were compared to trachea-derived ALI cultures. Cortisol withdrawal for the final 2 days of ALI cultures influenced goblet cell density (*p* = 0.001). Conclusions: While the histology score provides less resolution than FACS- or OMICs- based single cell analyses, the use of a subportion of the ALI epithelia grown on inserts makes it feasible to combine morphology assessment and other readouts of the same insert. This allows us to control for basic ALI morphology in research and personalized medicine settings in order to assess and, if desired, control for the impact of ALI culture protocols, site- and donor-specific influences on outcome of studies of ALI-derived epithelia.

## 1. Introduction

Respiratory diseases can be studied using primary epithelia grown at air–liquid interface (ALI) from patients’ biomaterials [1]. ALI epithelia have been described using nasal brushings [2,3,4,5], nasal biopsies [6], bronchial biosamples [5,7], explanted lung tissue [1,8,9], nasal polyps [10,11] or stem cells [12,13,14] as sources to regenerate primary epithelia. To generate primary epithelia, the airway epithelium continuity of the patient’s biomaterial needs to be destroyed and the presence of primary airway progenitor cells in the biomaterial needs to be maintained to achieve cell expansion. Finally, the re-differentiation on inserts at the air–liquid interface of those progenitor cells into primary epithelia is determined by their capability for regeneration [15,16].

Primary ALI epithelia have been used to characterize human diseases such as the monogenic diseases primary cilia dyskinesia [12] and cystic fibrosis [3,5,12] as well as complex diseases such as asthma [7,17] and chronic obstructive pulmonary disease [8,18]. ALI epithelia are widely employed to test drugs [19,20,21] and to understand environmental influences [22,23], host defense [24,25] or genetic variants [3,10]. While such studies can be carried out using immortalized epithelial cell cultures, such cultures are monotypic and thus cannot fully represent the airway epithelium [26]. In contrast, ALI cultures from patient-derived biomaterials will display the typical basal, ciliated and goblet cell types as well as rare cell species such as ionocytes [27,28].

The variability of ALI culture outcome is partly determined by culture technique, especially as all protocols include the presence of cell differentiation factors or anti-inflammatory agents [26]. Moreover, when human biosamples from different donors are compared, the resulting experimental variability increases as ALI epithelia represent patient-to-patient dissimilarity in phenotypes. Of note, the latter variation comparing ALI epithelia is often the one addressed in personalized medicine.

To diminish the variabilities in ALI-based studies, we have devised an easy-to-apply histological scoring which helps qualify the morphological phenotype of each ALI insert. As only a small fraction of an ALI insert is needed for histology analyses [29], in addition to insert phenotyping via other techniques such as protein or transcript analysis, ALI morphology phenotyping can be accomplished using the same insert. By quantifying four key parameters in our ALI epithelia derived from explanted lung tissue (small airway epithelial cells, or SAEC [9]) or from the main (primary) bronchus (human bronchial epithelial cells, or HBEC), we are able to show that firstly, SAEC (derived from end-stage lung disease) and HBEC (derived from donor lung tissue) differ phenotypically and that cortisone alters ALI appearance. Both of these effects are well-known, and they demonstrate that the fast scoring of human primary respiratory ALI epithelia can detect these altered morphological characteristics faithfully and lends itself to the control of these known confounders. We propose a scoring system as a useful tool to monitor epithelial morphology on an insert-by-insert basis to control for method-to-method and/or patient-to-patient variability in research settings as well as to describe basic morphological properties of the ALI epithelia in a disease context and for personalized medicine.

## 2. Materials and Methods

Primary airway epithelia were reconstituted from explanted lung tissue or donor trachea as small airway epithelial cells (SAEC) or human bronchial epithelial cells (HBEC), respectively. During the period of 2019 to 2023, 16 SAEC and 38 HBEC cultures were derived with a total of 106 inserts. The study was approved by the Medical Ethics Committee of the Hannover Medical School (#2699-2015). The generation of ALI epithelia was based on protocols from Muller et al. [6], Fulcher et al. [1] and Zarcone et al. [8], creating a synthesized protocol described in detail elsewhere [29]. Briefly, ALI culture can be described as a three-stage-process of isolation of primary airway progenitor cells, followed by an expansion culture, followed by seeding the expanded cells on inserts and allowing differentiation at the air–liquid interface [1,6,8]. For cell isolation from explanted lung tissue, parenchymal tissue was removed from smaller airways. Smaller bronchi or trachea were cut open to expose the epithelium and subjected to protease digestion. Next, cells were scraped from the surface of the bronchi or trachea with the backside of a scalpel and expanded in a modified keratinocyte medium [8] in T25 flasks. When confluency was reached adherent cells were detached in a two-step protocol [1] and seeded in a cell density of 2.5 to 3.5 × 10^5^ cells per 200 µL of BEGM per insert on a 12-well ALI plate (0.4 µm pore polyethylene terephthalate membrane inserts) with 720 µL BEGM ALI medium provided basolaterally. Upon confluency, the apical medium was completely removed. Epithelial cell layers were allowed to differentiate at ALI with Pneumacult ALI medium (Stemcell technologies, Vancouver, BC, Canada) for at least three weeks until cilia beating could be observed under the microscope.

For this study, histology samples were generated as follows: All inserts were divided to provide readout for protein, RNA and histology in parallel for each insert [29]. For the latter, a fifth to a sixth of each the 12-well inserts was used for histology analysis, dividing the small insert section again into two factions [29]. One of these was used for classical formamide fixation and, in parallel, a methacarn fixation was employed to preserve mucus structures. For this, ALI cultures were fixed in 4% buffered formaldehyde for 24 h or for methacarn fixation, ALI cultures were incubated with 60% methanol, 30% chloroform and 10% acetic acid for 24 h, respectively. In the context of this work, histology scores were mostly derived from formamide fixed sections. Next, following standard histological procedures [29], these fixated ALI epithelia were embedded in paraffin and sectioned into 2 μm thick slices.

For the embedding procedure, fixated ALI inserts were rinsed twice with 100% ethanol for 30 min each and twice with xylene for 30 min each. The ALI inserts were then placed sandwiched between two embedding cassette filter pads in a tissue embedding cassette and embedded in paraffin wax. As this would leave the orientation of the primary epithelia perpendicular within the cassette when mounting them into the microtome, leading to cross-sectional cuts through the epithelial layers from top to bottom, ALI epithelia were rotated after this embedding stage. For that, wax-embedded ALIs were retrieved from the tissue embedding cassette; filter pads were removed; and embedded inserts were rotated by 90°, replaced in a tissue embedding cassette and re-embedded into paraffin wax prior to mounting in the microtome.

The 2 µM sections of ALI epithelia were dewaxed and afterwards stained with hematoxylin-eosin in the Multistainer 3 (device designation: TST 40 Fa. Medite, Burgdorf, Germany) utilizing the following method: treatment was done with xylene for 5 min and a further 90 s, 100% ethanol for 90 s, 90% ethanol for 90 s, 70% ethanol for 90 s and, finally, deionized water for 90 s. Next, staining was carried out with hemalum solution for 4 min, deionized water for 1 min, 70% ethanol with 1% hydrochloric acid for 10 s and deionized water for 3 min. Finally, eosin staining was carried out for 3 min and destaining was performed with deionized water for 1 min and 90% ethanol for 90 s, 100% ethanol twice for 90 s each and, finally, xylol for 90 s.

The histological scoring of slides was carried out using an Olympus microscope with five different augmentation objectives (2×/0.06; 4×/0.10; 10×/0.25; 20×/0.40; 40×/0.65), investigating ALI epithelia for differentiation into respiratory epithelium and qualifying the number of cell layers (layer score), the occurrence of apoptosis (apoptosis score), air inclusion structures (air inclusion score) and goblet cells (goblet cell score). For each, a set of visual analogue scales was used to score the ALI epithelium (Supplementary Figure S1). In detail, the insert preparations were examined along their entire length and the following criteria were used to assign scores from 0 to 3 for each of the four ALI phenotypes. The layer height of the epithelium was scored using the nuclei of the epithelial cells as orientation, assigning a layer score of 0 to ALI epithelia with 1 to 2 rows of cells on top of each other, a layer score of 1 to ALI epithelia with 3 to 5 rows of cells, a layer score of 2 for ALI epithelia with 5–7 rows of cells and a layer score of 3 to ALI epithelia with even more cell layers. Next, apoptoses were recognized by their disintegrating or fragmented cell nuclei in the initial stage or by the bright eosinophilic discoloration of the underlying cell in the later stages. An apoptosis score of 0 was assigned to ALI epithelia with no or only one solitary apoptosis while for an apoptosis score of 1, more than one apoptosis, but few in total, were observed. Epithelia with increased apoptosis were described with an apoptosis score of 2 and an apoptosis score of 3 were assigned to ALI epithelia with many apoptoses that were conspicuously arranged in larger groups. Furthermore, as we have occasionally observed voids within the ALI epithelia which were typically lined by epithelia showing apical differentiation features such as ciliated cells, we qualified such air inclusion structures. ALI epithelia that do not show such voids received an air inclusion score of 0, epithelia with only a single void in the entire preparation were scored 1 for air inclusion, and an air inclusion score of 2 indicated that there were few voids throughout the epithelium, while an air inclusion score of 3 was used for epithelia with several air inclusions in the examined tissue. Finally, we assessed goblet cell density, acknowledging the loss of the kinocilia due to the apically directed goblet cells. Epithelia without goblet cells were described with a goblet score of 0, ALI epithelia with few goblet cells were labeled with a goblet cell score of 1. A goblet cell score of 2 described a density of goblet cells on the surface that is close to 50 percent and epithelia with goblet cells that take up well over 50 percent of the epithelial surface were described with a goblet cell score of 3. To differentiate reliably between adjacent categories, the same set of visual analogue scales (Supplementary Figure S1) was used as a reference to assign histological scores to the ALI epithelia.

## 3. Results

### 3.1. Morphology of Epithelia Grown at the Air–Liquid Interface

For histology, we have used 106 inserts from 16 SAEC and 38 HBEC cultures derived from 54 different individuals. For eight of these 54 donors, ALI tissues could not be evaluated histologically, corresponding to a drop-out rate of 14.8%. Specifically, during the handling of the 106 ALI inserts for fixation and embedding, biomaterial from 31 inserts was partially or completely lost and could not be evaluated histologically, corresponding to a drop-out rate of 29%. This demonstrates that basic morphology characteristics can be obtained from most ALI epithelia on an insert-by-insert basis, using only a small portion of each insert, thus leaving most biomaterial intact for other analyses. In our experience, the critical step of this procedure is during the harvest of inserts when biomaterials are collected for the various readout procedures. The epithelia less rigidly attached to their inserts and/or inserts that are handled less carefully during the initial fixation step cannot be recovered for histology.

**Figure 1:**
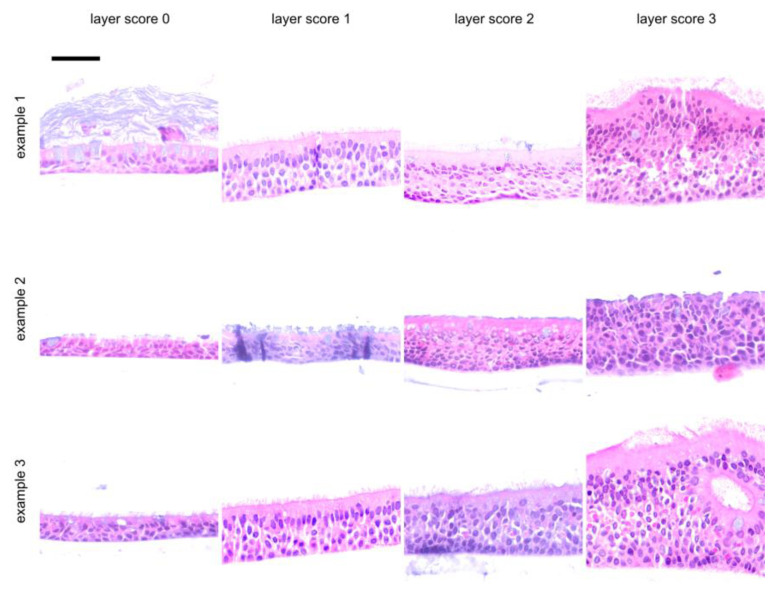
Representative examples for ALI layer score. These examples were selected from independent donor sources, except for examples 1 and 3 in layer score 1, which were derived from the same donor. Example 2 for a layer score of 2 was the only tissue which is fixed using methacarn, and all others were treated using formamide. Using a visual analogue scale (Supplementary Figure S1) as a reference, a layer score of 0 was assigned to 1–2 rows of cells, a layer score of 1 for 3–5 rows of cells, a layer score of 2 for 5–7 rows of cells and a layer score of 3 for thick epithelia with 7 or more rows of cells. The scalebar is 50 µm for all inserts.

**Figure 2:**
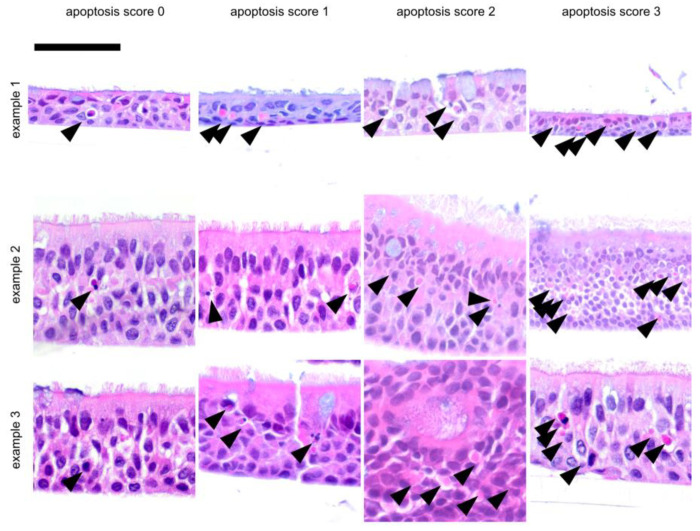
Representative examples for ALI apoptosis score. These examples were selected from independent donor sources, except for example 2 and 3 in layer score 0, which were derived from the same tissue. Arrows show apoptosis which can be recognized by their disintegrating or fragmented cell nuclei in the initial stage or by the bright eosinophilic discoloration of the underlying cell in the later stages. Using a visual analogue scale (Supplementary Figure S1) as a reference, an apoptosis score of 0 was assigned to ALI epithelia with no or only one solitary apoptosis. For an apoptosis score of 1, more than one apoptosis, but few in total, were observed. An apoptosis score of 2 indicated increased apoptoses, some of which were grouped together. An apoptosis score of 3 indicated many observable apoptoses that were conspicuously arranged in larger groups. The scalebar is 50 µm for all inserts.

**Figure 3:**
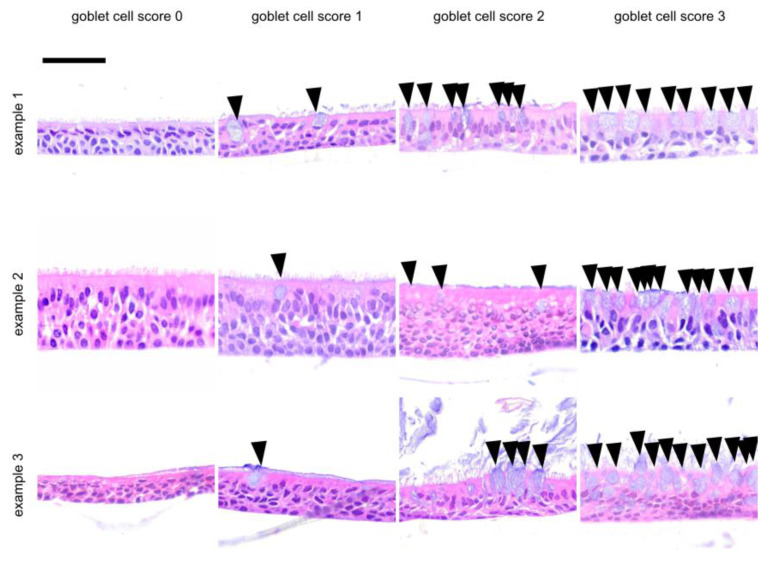
Representative examples for ALI goblet cell score. These examples were selected from independent donor sources. Arrows denote isolated goblet cells, which belong to the intraepithelial glandular cells and serve to protect the mucous membranes by producing mucus and secreting it apically. Finally, a mucin film is formed on the surface, which is continuously transported further via the ciliated epithelium (kinocilia). A frequently observed phenomenon is the loss of the kinocilia due to the apically directed goblet cells. Using a visual analogue scale (Supplementary Figure S1) as a reference, a goblet cell score of 0 was assigned to epithelia without goblet cells, a goblet score of 1 denotes a few goblet cells, whereas a goblet cell score of 2 was assigned to epithelia with a goblet cell density on the surface that was close to 50 percent. A goblet cell score of 3 was used to indicate well over 50 percent of goblet cells of the epithelial surface. The scalebar is 50 µm for all inserts.

**Figure 4:**
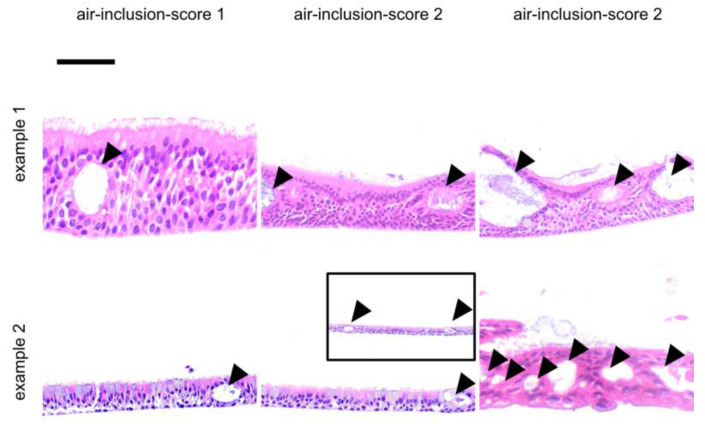
Representative examples for ALI air inclusion score. These examples were selected from independent donor sources. Arrows denote air inclusions in the cell layer. Using a visual analogue scale (Supplementary Figure S1) as a reference, an air inclusion score of 0 was assigned to ALI epithelia without air inclusions, an air inclusion score of 1 indicated that only one single void was observed in the in the entire preparation, an air inclusion score of 2 was attributed to ALI epithelia with few voids, while an air inclusion score of 3 was attributed to ALI epithelia with several air inclusions in the examined tissue. The scalebar is 50 µm for all inserts.

Scores were assigned to a total of 54 ALI epithelia derived from different donors. Slides were stained with hematoxylin and eosin (HE) and were investigated for epithelial layers (layer score, Figure 1), for the presence of apoptotic cells (apoptosis score, Figure 2) and goblet cells (goblet cell score, Figure 3) and for the inclusion of air within the epithelial layer (air inclusion score, Figure 4). Figure 1, Figure 2, Figure 3 and Figure 4 illustrate representative examples of each morphological phenotype.

To assess the repeatability and observer-dependency of our scoring method, we have compared all four score criteria between studies conducted from 2019 to 2021 (12 SAEC and 27 HBEC, study 1) and from studies conducted from 2022 to 2023 (4 SAEC and 11 HBEC, study 2). Two independent observers scored ALI epithelia in these two observation periods. As these studies were challenge-and-intervention experiments from two different research projects [29], only untreated control inserts were included in this evaluation to judge observer-to-observer independence. As shown in Table 1, score distributions were mostly comparable for both study periods. The most frequent morphologies were characterized by a layer score of 1, accounting for three out of four inserts; a goblet cell score of 1, accounting for roughly half of the inserts, and the absence of air inclusions, observed for about half of the inserts. All of these phenotypes were consistent in both studies (Table 1). The two studies disagreed most significantly on the apoptosis as half of the inserts in the earlier study (study 1) did not show apoptosis (apoptosis score of 0), while half of the inserts from the later study (study 2) had an apoptosis score of 1 (*p* = 0.03, Table 1).

### 3.2. Difference in Morphology of SAEC and HBEC Epithelia and the Effect of Cortisol Can Be Distinguished via the ALI Histology Score

To validate the ALI histology score, we stratified our data with respect to two research questions for which a difference in morphology can be expected based on published data. First, we compared the appearance of SAEC (derived from end-stage lung disease patients) and HBEC (derived from donor lung trachea) (Table 2), and second, we investigated the influence of cortisol-withdrawal during the final two days of ALI culture (Table 3).

Our data show a significant difference in layer score between SAEC and HBEC groups (*p* = 0.001, Table 2), with HBEC displaying more cell layers than SAEC. The occurrence of apoptosis also differed between SAEC and HBEC, although it did not reach statistical significance (*p* = 0.07, Table 2). The absence of apoptosis (apoptosis score of 0) was found more frequently in the SAEC group. There was a significant difference in goblet cell score between the two groups (*p* = 0.002, Table 2), with SAEC displaying more goblet cells than HBEC. Air inclusions were equally frequent in both subsamples (Table 2).

When comparing the ALI cultured using a standard procedure in the presence of cortisol throughout the entire ALI culture period to the ALI deprived of cortisol in the last two days of culture, the thickness of ALI epithelia was significantly different—thicker ALI epithelia were observed when cortisol was withdrawn for 2 days prior to harvest (*p* = 0.01, Table 3). Moreover, ALI epithelia grown continuously with cortisol had a higher apoptosis score (*p* = 0.04, Table 3). Cortisol withdrawal had the highest impact on goblet cell density as significantly more goblet cells were observed in ALI continuously grown in the presence of cortisol (*p* = 0.001, Table 3). Again, subsamples did not differ with respect to air inclusions (*p* = 0.1, Table 3).

## 4. Discussion

Examples of recent publications on the use of air–liquid interface cultures are complex functional studies on the role of the IgE receptor [31] or rhinovirus infection [32,33] in ALI epithelia derived from patients’ bronchial biopsies [31,32] or nasal brushings [33] in the context of asthma, as well as patient-centered approaches to provide biomaterial for individual drug testing in the context of cystic fibrosis [19]. Of note, personalized medicine requiring that the patients’ biomaterials are studied prior to off-label prescription has been realized already for the multiallelic disease cystic fibrosis [34], and patient-derived ALI epithelia are used prior to therapeutic decisions in cystic fibrosis, an approach referred to as theratyping [35,36,37].

In recent years, of more complex ALI protocols involving minimal, cortisol-free media [38] or starvation [32] prior to challenge experiments have been established. At the same time, studies with few patients involving heterogeneous sampling techniques have been conducted. Together, these two settings necessitate knowledge on the morphological structure of the primary ALI epithelium to take into account the large heterogeneity typically observed in ALI cultures, which is exacerbated by different treatment protocols, sampling locations and donor-specific variabilities.

For instance, it appears highly relevant to distinguish whether case and reference ALI biomaterials already differ at the starting point of a functional study or whether such epithelia are similar, despite reacting differently to the stimulus investigated in the functional study. The former would indicate that cases and controls differ in their capabilities to regenerate epithelia, reflecting their endophenotypes, while the latter would confirm that epithelia differ on a functional level. Of note, even though iPSCs provide a reliable and renewable source of basal cells and/or primary airway progenitor cells, even with such a homogenous cell source to grow ALI epithelia from, morphological and functional differences for iPSC-derived ALI are observed depending on the culture protocol [39].

Cellular morphology on a case-by-case, insert-by-insert basis is usually not at the focus of the studies where the desired readout is the transcriptome or the cellular or secreted proteome (or parts thereof). Thus, we propose the fast scoring of human primary respiratory ALI epithelia to assess epithelial morphology in research and personalized medicine settings by using only a small portion of an ALI insert. We were able to demonstrate that our score (Figure 1, Figure 2, Figure 3 and Figure 4) is robust and observer-independent (Table 1) but sensitive enough to detect morphological differences between SAEC (derived from end-stage lung disease patients) and HBEC (derived from donor lung trachea) (Table 2) as well as cortisol withdrawal after ALI differentiation (Table 3). As only a minor part of the insert is devoted to histology scoring, other readouts from the ALI epithelia can be obtained in parallel, enabling more complex studies than merely a low-resolution histology score. This provides the opportunity to compare case and reference samples with similar morphology in studies that require the accumulation of multiple ALI epithelia from different donors. Conversely, a small set of donor-dependent ALI epithelia used to study a disease entity or a specific treatment condition will help to find donor-dependent alterations of cellular composition, which can be directly correlated to the readout of such studies. Based on our real-world experience with the technique, involving trained albeit changing personnel within a 4-year time period, we feel that even acknowledging the drop-out rate of 15% (based on donor tissue count) or 30% (based on insert count), our study has benefited considerably from our morphology analysis, as we would otherwise have been ignorant of the influence of cortisol withdrawal on the ALI phenotype.

Despite easy-to-obtain morphology characteristic for ALI epithelia, our study has several limitations: the histology score is much less complex and has a much lower resolution than OMICs-guided methods such as single cell sequencing or fluorescence-activated cell sorting. Moreover, our ALI morphology analyses have been conducted as a retrospective study, building on ALI material from different research projects, whereby the donor material or the cell culture protocols were adjusted based on a research question. Finally, if ALI inserts with a smaller diameter than the 12-well format are used, researchers might have to prioritize their readouts. In this regard, some measurements are compatible and can be combined, such epithelial electrophysiology using a micro ussing chamber and subsequent protein analysis, which demonstrates that the morphology of the epithelium can be assessed in parallel to such functional data [40].

In summary, the rapid assessment of human primary respiratory ALI epithelia to assess epithelial morphology can be achieved using only a portion of an ALI epithelium grown on 12-well plate inserts, allowing the control of basic ALI morphology in research and personalized medicine settings for which more complex—and often more expensive—readouts are desired. Finally, assessing the morphology suggested in our study will allow researchers to assess laboratory-to-laboratory differences in culture protocols or donor cohorts. This would improve reproducibility between studies from different research groups or make it feasible to judge the reason for a lack thereof between studies from different authors.

## Figures and Tables

**Table 1:** Observer-to-observer independence of histology score distribution for two time periods when different observers scored ALI epithelia.

Score	Layer		Apoptosis		Goblet Cell Score		Air Inclusion	
Data Set	Study 1 ^1^	Study 2 ^2^	Study 1 ^1^	Study 2 ^2^	Study 1 ^1^	Study 2 ^2^	Study 1 ^1^	Study 2 ^2^
0	5 [10.9%]	6 [21.4%]	19 [42.2%]	4 [14.3%]	4 [9.1%]	7 [25.9%]	23 [52.3%]	16 [59.3%]
1	34 [73.9%]	19 [67.9%]	8 [17.8%]	14 [50%]	30 [68.2%]	10 [37.0%]	15 [34.1%]	9 [33.3%]
2	4 [8.7%]	2 [7.1%]	14 [31.1%]	9 [32.1%]	6 [13.6%]	3 [11.1%]	5 [11.3%]	2 [7.4%]
3	3 [6.5%]	1 [3.6%]	4 [8.9%]	1 [3.6%]	4 [9.1%]	7 [25.9%]	1 [2.3%]	0 [0%]
*p* ^3^	1		0.03		1		0.9	

^1^ from studies conducted from 2019 to 2021 (12 SAEC and 27 HBEC). ^2^ from studies conducted from 2022 to 2023 (4 SAEC and 11 HBEC). ^3^ computed using a Monte Carlo simulation of the kx2 table using the algorithms of Sham and Curtis [30].

**Table 2:** Histology score distribution for 41 SAEC and 65 HBEC.

Score	Layer		Apoptosis		Goblet Cell Score		Air Inclusion	
Data Set	SAEC	HBEC	SAEC	HBEC	SAEC	HBEC	SAEC	HBEC
0	10 [33.4%]	1 [2.3%]	13 [43.3%]	10 [23.2%]	2 [6.9%]	9 [21.4%]	16 [55.2%]	23 [54.8%]
1	18 [60%]	35 [79.5%]	8 [26.7%]	14 [32.6%]	12 [41.4%]	28 [66.7%]	9 [31.0%]	15 [35.7%]
2	1 [3.3%]	5 [11.4%]	8 [26.7%]	15 [34.9%]	6 [20.7%]	3 [7.1%]	4 [13.8%]	3 [7.1%]
3	1 [3.3%]	3 [6.8%]	1 [3.3%]	4 [9.3%]	9 [31.0%]	2 [4.8%]	0 [0%]	1 [2.4%]
*p* ^1^	0.001		0.07		0.002		0.67	

^1^ computed via the Monte Carlo simulation of the kx2 table using the algorithms of Sham and Curtis [30].

**Table 3:** Histology score distribution for ALI epithelia generated continuously with cortisol (27 SAEC; 35 HBEC) or without cortisol during the last two days of culture prior to harvest (10 SAEC; 21 HBEC).

Score	Layer		Apoptosis		Goblet Cell Score		Air Inclusion	
Cortisol	YES ^1^	NO (2d) ^2^	YES ^1^	NO (2d) ^2^	YES ^1^	NO (2d) ^2^	YES ^1^	NO(2d) ^2^
0	6 [14.3%]	5 [15.6%]	13 [31.7%]	10 [21.3%]	2 [4.9%]	9 [30%]	23 [56.1%]	16 [53.3%]
1	34 [80.9%]	19 [59.4%]	9 [21.9%]	13 [40.6%]	22 [53.7%]	18 [60%]	15 [36.6%]	9 [30%]
2	0 [0%]	6 [18.8%]	17 [41.5%]	6 [18.7%]	8 [19.5%]	1 [3.3%]	2 [4.9%]	5 [16.7%]
3	2 [4.8%]	2 [6.2%]	2 [4.9%]	3 [9.4%]	9 [21.9%]	2 [6.7%]	1 [2.4%]	0 [0%]
*p* ^3^	0.01		0.04		0.0001		0.1	

^1^ standard protocol for ALI culture whereby cortisol is included in the medium continuously. ^2^ cortisol was withdrawn from the medium 2 days prior to harvest. ^3^ computed via the Monte Carlo simulation of the kx2 table using the algorithms of Sham and Curtis [30].

## Data Availability

Source data are provided as supplementary information with this manuscript.

## Supplementary Materials

The following supporting information can be downloaded at: https://www.mdpi.com/article/10.3390/jpm14010109/s1. Figure S1: Visual analogue scales to score ALI epithelia.
