# Supplementary material for: A Fast Scoring of Human Primary Respiratory Epithelia Grown at Air–Liquid Interface (ALI) to Assess Epithelial Morphology in Research and Personalized Medicine Settings
Source: J Pers Med. 2024 Jan 18;14(1):109. doi: 10.3390/jpm14010109 (PMC10817428; doi:10.3390/jpm14010109)
Supplement: Supplementary file 1 [file jpm-14-00109-s001.zip › jpm-2769184-supplementary.pdf]

Supplementary material for:

A fast scoring of human primary respiratory epithelia grown at air-liquid-interface (ALI) to assess epithelial morphology in research and personal medicine settings

Christopher Lutsch [1,2 ‡], Longhua Feng [1,2 ‡], Ana Gomez-Hohn[3‡], Lennart Brandt[3], Stephanie Tamm[1,2], Sabina Janciauskiene[4,2], Frauke Stanke[1,2], Danny Jonigk[2,5], Anna-Maria Dittrich[1,2] and Peter Braubach[3,*]

1 Department of Pediatric Pneumology, Allergology and Neonatology, Hannover Medical School, Hannover, Germany
2 Biomedical Research in Endstage and Obstructive Lung Disease Hannover (BREATH), German Center for Lung Research (DZL), Hannover, Germany
3 Institute for Pathology, Hannover Medical School, Hannover, Germany
4 Department of Respiratory Medicine, Hannover Medical School, Hannover, Germany
5 Institute of Pathology, School of Medicine, University Hospital RWTH Aachen, Aachen, Germany

* Correspondence: Braubach.Peter@mh-hannover.de;

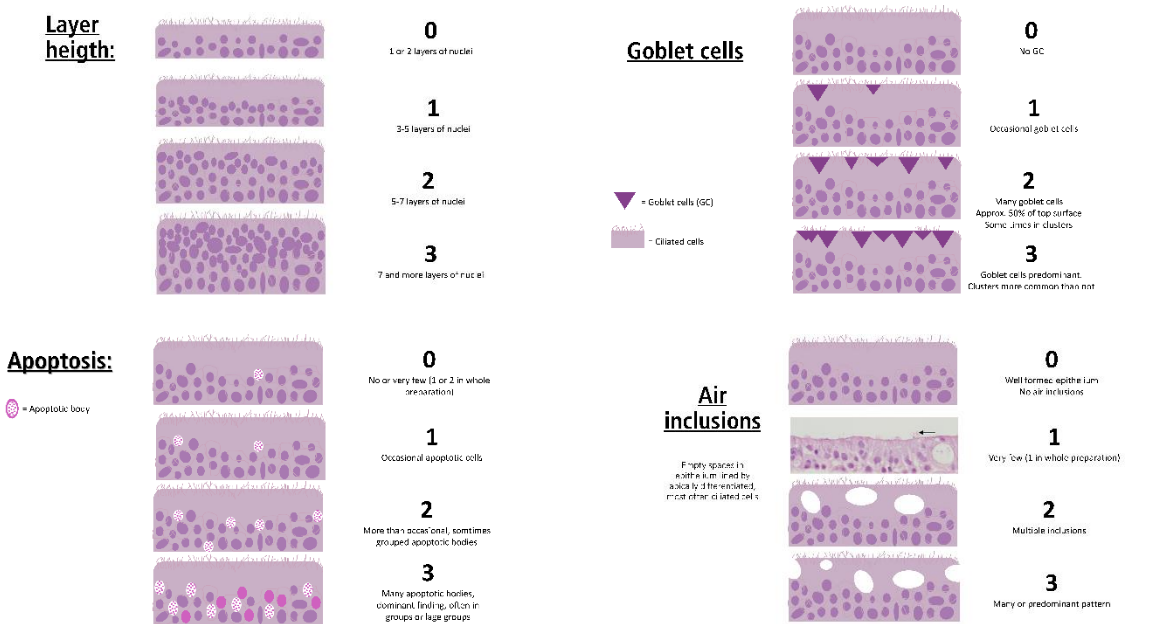

Supplemental Figure S1. Visual analogue scale for scoring of ALI epithelia. These 4 panels show the visual analogue scales used to score the ALI epithelia for layer height (top left), goblet cells (top right), apoptosis (bottom left) and air inclusions (bottom right). As outlined graphically, the following criteria were used to assign scores from 0 to 3 for each ALI phenotype: **Layer height score**: The layer height of the epithelium was evaluated using the nuclei of the epithelial cells as orientation. 1 to 2 rows of cells on top of each other defined a layer score of 0. If 3 to 5 rows of cells were arranged, the layer was rated with a layer score of 1. If there were 5-7 rows of cells, a layer height score 2 was assigned. For 7 or more rows of cells, the layer height score was 3. **Goblet cell score:** Goblet cells belong to the intraepithelial glandular cells and serve to protect the mucous membranes by producing mucus and secreting

it apically. Finally, a mucin film is formed on the surface, which is continuously transported further via the ciliated epithelium (kinocilia). A frequently observed phenomenon is the loss of the kinocilia due to the apically directed goblet cells. For the goblet cell score, 0 means no goblet cells are seen, 1 means "very few" goblet cells can be seen, goblet cell score 2 describes a density of goblet cells on the surface that is close to 50 percent. The goblet cell score 3 was used to indicate "very many goblet cells", which make up well over 50 percent of the epithelial surface. **Apoptosis score:** The apoptoses can be recognized by their disintegrating or fragmented cell nuclei in the initial stage or by the bright eosinophilic discoloration of the underlying cell in the later stages. For the examiner, 0 means that there was no or only one solitary apoptosis in the epithelium. For apoptosis score 1, more than one apoptosis, but few in total were observed. Apoptosis score 2 described increased apoptoses, some of which were grouped together. Apoptosis score 3 indicated seeing many apoptoses that were conspicuously arranged in larger groups. **Air-inclusion – score:** We assume that the formation of air-inclusion, appearing as appearing as voids in the epithelium typically lined by epithelia showing apical differentiation features (e.g. cilia), is associated with preceding larger groups of apoptoses. Air inclusion score 0 indicates there was no air-inclusion in the lung epithelium, air inclusion score 1 means that only one single void was observed in the in the entire preparation. An air-inclusion score of 2 indicates that there were few voids throughout the epithelium. Air inclusion score 3 describes the finding of several air-inclusions in the examined tissue.
